# Short-Term Motor Outcomes in Parkinson's Disease after Subthalamic Nucleus Deep Brain Stimulation Combined with Post-Operative Rehabilitation: A Pre-Post Comparison Study

**DOI:** 10.1155/2022/8448638

**Published:** 2022-08-12

**Authors:** Kazunori Sato, Yoshihide Hokari, Eriko Kitahara, Nana Izawa, Kozo Hatori, Kaoru Honaga, Genko Oyama, Taku Hatano, Hirokazu Iwamuro, Atsushi Umemura, Yasushi Shimo, Nobutaka Hattori, Toshiyuki Fujiwara

**Affiliations:** ^1^Department of Rehabilitation Medicine, Juntendo University Graduate School of Medicine, Tokyo, Japan; ^2^Department of Rehabilitation Medicine, Juntendo University Nerima Hospital, Tokyo, Japan; ^3^Department of Rehabilitation Medicine, Juntendo University Urayasu Hospital, Tokyo, Japan; ^4^Department of Neurology, Juntendo University Hospital, Tokyo, Japan; ^5^Department of Research and Therapeutics for Movement Disorders, Juntendo University Graduate School of Medicine, Tokyo, Japan; ^6^Department of Neurosurgery, Juntendo University Hospital, Tokyo, Japan; ^7^Department of Neurology, Juntendo University Nerima Hospital, Tokyo, Japan; ^8^Department of Physical Therapy, Juntendo University Faculty of Health Science, Tokyo, Japan

## Abstract

**Background:**

The effects of subthalamic nuclear deep brain stimulation therapy (STN-DBS) and combined postoperative rehabilitation for patients with Parkinson's disease with postural instability have yet to be well reported. This study investigated the effects of short-term postoperative rehabilitation with STN-DBS on physical function in patients with Parkinson's disease.

**Methods:**

Patients diagnosed with Parkinson's disease who were admitted to our hospital for STN-DBS surgery were included in this study. Data were prospectively collected and retrospectively analyzed. Postoperative rehabilitation consisted of muscle-strengthening exercises, stretching, and balance exercises for 40–60 minutes per day for approximately 14 days. The Mini-Balance Evaluation Systems Test (Mini-BESTest), Timed Up and Go test (TUG) seconds and steps, Trunk Impairment Scale (TIS), seconds for 10 times toe-tapping, lower limb extension torque using StrengthErgo240, and center of pressure sway in the quiet standing posture were evaluated preoperatively, postoperatively, and at discharge. Mini-BESTest changes were also evaluated in the two groups classified by the presence or absence of postural instability. One-way and two-way repeated measures analyses of variance were performed for each of the three periods of change, and paired *t*-tests with the Bonferroni method were performed as multiple comparison tests. A stepwise multiple regression model was used to identify factors associated with balance improvement.

**Results:**

A total of 60 patients with Parkinson's disease were included, and there were significant increases in Mini-BESTest, TIS, StrengthErgo240, and postural sway during closed-eye standing compared to pre- and postoperative conditions at discharge (*p* < 0.05), and they decreased significantly compared to the postoperative period (*p* < 0.05). On stepwise multiple regression analysis, decreased steps of TUG and improvement of TIS scores were related to improvement of the Mini-BESTest (*p* < 0.05). In addition, Mini-BESTest scores in both groups with and without postural instability were significantly increased at discharge compared to preoperative and postoperative conditions (*p* < 0.01).

**Conclusion:**

Postoperative rehabilitation combined with STN-DBS may provide short-term improvements in physical function compared with the preoperative medicated status. The improvements in gait step length and trunk function may be important factors for obtaining improvement of postoperative postural stability.

## 1. Background

Parkinson's disease (PD) is a neurodegenerative disease characterized by slowly progressive motor and nonmotor impairments caused by decreased dopamine neurons in the substantia nigra of the midbrain. In addition to the three major signs of resting tremor, muscle rigidity, and bradykinesia, PD patients present with a variety of manifestations, including postural instability and cognitive decline in the advanced stages of the disease [1, 2]. Since a quarter of a century ago, subthalamic nuclear stimulation therapy (STN-DBS) has been adopted worldwide to reduce motor complications and wearing-off symptoms in PD patients [3–6]. However, previous studies that reported the effect of STN-DBS on postural instability were limited. Some observational studies showed that STN-DBS did not improve postural stability after surgery compared to before on-medication status [7, 8]. The randomized, controlled trials showed that the effect of STN-DBS on balance function did not exceed the best-medicated state before surgery [3, 8–11]. In contrast, a few studies reported the effectiveness of STN-DBS for postural instability and postural deformity. A retrospective study suggested that the STN-DBS was effective for improving motor disability and balance performance [12]. Another retrospective study indicated the positive effect of STN-DBS for postural alignment [4]. A few studies, although very limited in number, have investigated the impact of combined post-operative rehabilitation therapy after STN-DBS [13–15]. Previous studies have reported that postoperative rehabilitation in conjunction with stimulation adjustment after STN-DBS improves activities of daily living (ADL), gait, and balance function. However, the improvement in balance function is still limited to cases of mild postural instability [13, 15], and which physical functions contribute to the improvement in postural instability has not been considered. Several studies have shown that background factors that contribute to balance function include trunk function, lower extremity muscle strength, bradykinesia, and walking ability [16–20]. Therefore, the following two points were examined in the present study. First, the effects of postoperative rehabilitation combined with STN-DBS on balance function in PD patients were examined. Which background factors (trunk function, gait speed, step counts, lower extremity bradykinesia, and lower extremity muscle strength) may be associated with changes in balance function was also investigated. Second, how balance function, which was considered to be poorly improved by STN-DBS, changed when patients were divided into two groups based on the presence or absence of postural instability before surgery was also examined.

## 2. Methods

### 2.1. Research Design

A pre- and postcomparison study was conducted at a single acute care hospital in Tokyo. The data were collected prospectively and analyzed retrospectively. Consecutive PD patients admitted to our hospital for STN-DBS surgery were included in the study. Based on the sample size calculation, the target number of patients was 66, when the results of each assessment parameter were assumed to be compared among the three groups of before, after, and at the time of discharge, with an effect size of 0.25, a significance level of 0.05, and power of 0.95. This study procedure was conducted with the approval of the ethics committee of Juntendo University Hospital (JHS18-276).

### 2.2. Study Subjects

Consecutive patients admitted to the Juntendo University Hospital between March 1, 2017, and December 31, 2018, for STN-DBS surgery and who underwent rehabilitation after surgery were included. The exclusion criteria were as follows: (1) those with complications (orthopedic diseases, such as osteoarthritis, or medical diseases, such as heart failure) that significantly reduced physical functioning before surgery; and (2) those who developed serious psychiatric symptoms as a complication of the surgery itself or who presented with delirium after surgery.

An interdisciplinary team including neurologists and neurosurgeons specialized in movement disorders evaluated the indications for STN-DBS a few months before STN-DBS surgery in all cases. The indications for STN-DBS followed our criteria [21]: (1) a clinical diagnosis of clinically established or clinically probable PD [1]; (2) severe diurnal fluctuations despite appropriate medication (e.g., dopamine-induced dyskinesia, wearing-off phenomenon, and on-off phenomenon) or inability to sufficiently increase the L-dopa medication dose due to side effects; (3) a good response to L-dopa medications (>30% improvement on the L-dopa challenge test); (4) no cognitive decline or psychiatric symptoms (Mini-Mental State Examination scores >24/30); (5) consented to and requested surgery; (6) no complications affecting electrode implantation (previous neurosurgery, tumors, calcification, etc.); (7) no cardiac pacemaker treatment; (8) an ability to tolerate general anesthesia; and (9) age less than 70 years desirable (age 70 years or older evaluated on an individual basis).

### 2.3. Postoperative Rehabilitation

Postoperative rehabilitation began on the third day after STN-DBS implantation surgery, and all patients received physical therapy for 40–60 minutes per day for approximately 14 days. The therapeutic intervention was carried out by a physical therapist skilled in movement disorders and was based on the neurological physical therapy guidelines for PD. It consisted of a general physical therapy program that included a combination of muscle-strengthening exercises, stretching, and balance exercises. STN-DBS stimulation began approximately 7 days after surgery, and the L-dopa medication dose was reduced as the intensity of the current increased and replaced the efficacy of the L-dopa medication. Approximately 2 weeks after surgery, the neurologist and neurosurgeon adjusted the amount of stimulation and dose reduction.

### 2.4. Clinical Assessment

At the time of admission for evaluation, the neurologist performed the L-dopa challenge test and assessed part III of the Movement Disorder Society-Unified Parkinson's Disease Rating Scale (MDS-UPDRS) motor items at the Off-state and Highest On-state. At the time of admission for surgery, physical function assessments were performed by a physical therapist at three points: preoperatively, three days after surgery, and immediately before discharge. All physical function assessments were performed between 60 and 120 minutes after oral medication, which is considered to be the On-state for PD medication.

#### 2.4.1. Mini-Balance Evaluation Systems Test

The Mini-Balance Evaluation Systems Test (Mini-BESTest) is a balance assessment test reported by Franchignoni et al. in 2010 that has been widely used around the world [22]. This test consists of four different balance items (anticipatory postural adjustment, reactive postural control, sensory integration, and dynamic gait) to comprehensively evaluate standing balance and gait function. The lowest score is 0, and the highest scored is 28, with higher scores indicating higher balance ability. In recent years, it has become a commonly used balance assessment test for PD patients because it is less likely to produce a ceiling effect in assessing balance function in PD patients and can predict falls [23, 24].

#### 2.4.2. Timed “Up and Go” Test

Gait function was assessed by the Timed “Up and Go” Test (TUG) used during the Mini-BESTest and the TUG-cognitive (TUG-cog) seconds and steps, respectively, in which the TUG is performed with a cognitive task [25]. The TUG is widely used worldwide to assess gait function in various neurological diseases, and more recently, it has been widely used to assess gait function in PD patients [26].

#### 2.4.3. Trunk Impairment Scale

The Trunk Impairment Scale (TIS) was reported in 2004 by Fujiwara et al. and was created to assess trunk function in stroke [27]. It consists of seven different items (vertical axis perception, left-right rotator strength, right-right turn reflex, left-right righting reflex, verticality, and forward abdominal muscle strength) and can assess various characteristics of the trunk individually.

#### 2.4.4. Evaluation of General Lower Extremity Extension Torque Using the StrengthErgo240

General lower limb extension muscle strength (Newton-meter) was evaluated using the StrengthErgo240 (SE240 : Mitsubishi Electric Engineering Corporation, Tokyo, Japan) [28]. Measurements were made in isokinetic mode with five consecutive drives at a rotational speed of 50 rotations/minute, and the peak left-right extension torque was measured during the lower limb's extension movement. The backrest angle was set at 110°, and the seat position was set, so that the knee joint was at 30° flexion and the ankle joint was at 0° dorsiflexion during maximum unilateral lower limb extension. Measurements were taken as the average of the right and left lower limb extension muscle forces.

#### 2.4.5. Lower Limb Bradykinesia Test (10 Toe-Tapping Seconds)

Lower extremity bradykinesia was assessed with MDS-UPDRS part III, item 7, toe-tapping (10 taps, as large and as fast as possible, with the toe) [29], and the seconds of tapping was measured. The measurements were averaged over the left and right sides.

#### 2.4.6. Postural Sway Test

Postural sway during opening and closing of the eyes in the standing posture was measured using Noraxon's myoPressure™ (Noraxon Inc., USA). The center of pressure (COP) 95% range circle, COP path length, and COP mean velocity were measured during both eye-opened and eye-closed standing postures for 30 seconds.

#### 2.4.7. Levodopa Daily Dose and Levodopa Equivalent Daily Dose

The levodopa daily dose (LDD) and levodopa equivalent daily dose (LEDD) were recorded based on the worldwide conversion method proposed by Tomlinson et al. to ensure the appropriateness of STN-DBS therapy [30].

### 2.5. Statistical Analysis

#### 2.5.1. Statistical Analysis for Study 1

The Mini-BESTest, TUG, TIS, SE240, 10 toe-tapping in seconds, gravity sway during eye-opening/closed standing, LDD, and LEDD at three time-points (before surgery, three days after surgery, and just before discharge) were calculated, and one-way repeated measures analysis of variance (ANOVA) was used to detect significant differences among the three 3 temporal moments. If there were significant differences, Bonferroni's paired *t*-tests were performed as post hoc multiple comparison tests. The correlations between the Mini-BESTest and other changed parameters of the assessments were analyzed by Spearman's correlation coefficients. The variance inflation factor was calculated to detect multicollinearity among dependent variables. After the determination of multicollinearity, multiple regression analysis was used to identify which improvements of physical functions were predictors of improvement of Mini-BESTest scores. The improvements were defined as the differences between the before surgery and the discharge-period scores.

#### 2.5.2. Statistical Analysis for Study 2

To analyze therapeutic effects in the patients with PD both who have postural instability or not before operation, number 12 of the MDS-UPDRS part III, postural stability item, were performed at the time of the assessment admission. A score of 3 or more on the PS item in the MDS-UPDRS indicates the absence of postural response [29]. According to this classification, we divided the patients into two groups: those with a postural stability score of 3 or more (with moderate or greater postural instability) and a mild case group with less than 3 points. Welch's *t*-test was used to test for significant differences between the two groups in basic information. In addition, the Mini-BESTest scores of the two groups were measured at three time-points (preoperatively, three days after surgery, and just before discharge) to detect significant differences among the three groups by two-way repeated measures ANOVA, and if there was a significant difference, post hoc paired *t*-tests using the Bonferroni method were performed as multiple comparison tests.

All statistical analyses were performed using the statistical software R (ver. 3.6.2), and the significance level was set at *p* < 0.05. Randomly occurring missing values were complemented by the multiple imputation method.

## 3. Results

Sixty-six patients were included in the study. Five patients were excluded due to postoperative delirium or worsening of psychiatric symptoms, and one patient was excluded due to complications of spinal canal stenosis, which had a strong impact on physical function. Thus, 60 of the 66 patients were included in this study.  Table 1 shows the demographic data of the total eligible cases and the basic attributes of the MDS-UPDRS part III postural stability items at the time of admission for evaluation, with the cases divided into those with a score of 3 or more and a score of less than 3.

### 3.1. Results of Study 1

The results of each clinical assessment are shown in Table 2. On ANOVA, there were significant differences in Mini-BESTest, TUG steps, TUG-cog steps, TIS, 10 toe taps, SE240, gravitational sway during closed-eyed standing, and LEDD. There were no significant differences in gait speed or center of gravity sway during open-eyed quiet stance. Multiple comparisons of the above clinical assessments, which were significantly different by ANOVA analysis, were performed using the Bonferroni method of paired *t*-tests, and the results showed that Mini-BESTest, TIS, SE240, 95% circle of the center of gravity (COP) during the closed-eyed stance, COP pass length, and COP mean speed were significantly increased at discharge compared to preoperative and postoperative; in TUG/TUG-cog steps, LDD, and LEDD, there were significant decreases at discharge status compared to preoperative and postoperative; in 10 toe taps, there was a significant decrease in time at discharge compared to preoperative and postoperative. Spearman's correlation coefficients showed the correlations between the Mini-BESTest and TUG seconds (*r* = −0.54, *p* < 0.01), TUG steps (*r* = −0.54, *p* < 0.01), TUG-cog seconds (*r* = −0.51, *p* < 0.01), TUG-cog steps (*r* = −0.41, *p* < 0.01), TIS (*r* = 0.25, *p*=0.05), 10 toe taps (*r* = −0.04, *p*=0.79), SE240 (*r* = 0.06, *p*=0.63), eye-opened COP 95% range circle (*r* = 0.20, *p*=0.11), eye-opened COP path length (*r* = 0.35, *p* < 0.01), eye-opened COP mean velocity (*r* = 0.34, *p* < 0.01), eye-closed COP 95% range circle (*r* = 0.21, *p*=0.09), eye-closed COP path length (*r* = 0.29, *p*=0.03), and eye-closed COP mean velocity (*r* = 0.33, *p* < 0.01). On multiple regression analysis, decreased steps of TUG and increased TIS scores were identified as predictors of improvement on the Mini-BESTest (*p* < 0.05) in Table 3.

### 3.2. Results of Study 2

**Figure 1:**
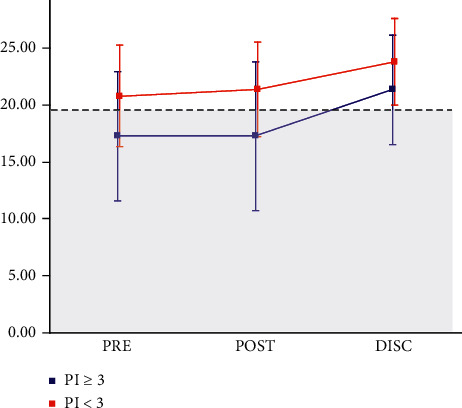
Mini-BESTest score changes in patients with and without postural instability. ^*∗*^gray field indicates the risk of falls (mini-BESTest < 19 points). abbreviations: mini-BESTest, mini-balance evaluation systems test; PI, the point of movement disorder society unified Parkinson's disease rating scale-part III 12 (postural stability score); PRE, preoperation period; POST, postoperation period; DISC, discharge period.

Two-way repeated ANOVA showed significant differences in the Mini-BESTest in both groups (*p* < 0.01), with no interaction (*p*=0.41). Multiple comparisons with the Bonferroni method of paired *t*-tests showed a significant increase in condition at discharge from the hospital in both groups compared to preoperative and postoperative (*p* < 0.01) (Figure 1).

## 4. Discussion

In this study, we observed the combined effect of STN-DBS and postoperative acute rehabilitation on balance function, which had not been previously clarified, and what these changes contributed to, as well as whether there was a combined effect of STN-DBS with postoperative rehabilitation on patients with PD who had postural instability from before surgery, was investigated. The results suggested that patients with PD who underwent early postoperative rehabilitation after STN-DBS improved their On-state balance and gait function compared with those who received the best medication before surgery, and the balance function was also improved in the group of patients who had postural instability before surgery.

Previous studies reporting the effects of STN-DBS postoperative rehabilitation reported improvements in scores of the Unified Parkinson's disease rating scale, Activities of Daily Living, and balance function, but it is not clear what contributed to the improvements in balance function [13, 15]. In the present study, postoperative rehabilitation combined with adjustment of stimulation settings and medications improved balance function, trunk function, lower limb muscle strength, and the number of steps during gait. It has been reported that trunk function, lower limb muscle strength, and stride length are related to balance function in PD [16–19]. Therefore, it is necessary to take into account the possibility that improvements in trunk function, lower limb muscle strength, and stride length contributed to the improvement of balance function. Multiple regression analysis suggested that a decrease in the number of steps taken, especially during walking, and improvements in trunk function may contribute to the improvement of balance function. Therefore, in the short term, the improvement of walking stride and trunk function may contribute more to the improvement of balance function than the improvement of lower limb muscle strength.

According to a previous study that reported the effects of early postoperative rehabilitation after STN-DBS, the balance function and gait ability may be improved in the short term when the subject has postural instability [15], but the present study, regardless of the presence or absence of postural instability, showed the possibility of improvement in balance and gait functions. Therefore, even in the short term, combining rehabilitation with adjustment of stimulation and medication after STN-DBS surgery in all cases may bring some benefits. Indeed, the present result showed that the short-term improvement in the mean Mini-BESTest value was above the cutoff value (Figure 1 and Table 3), which is 19 points, as shown in our previous study [23]. In addition, the changes in the Mini-BESTest in the present study were also clinically meaningful compared to the previous study of neurological diseases including PD, which has been reported to be 4 points [31].

As stated in research reports investigating the mid-to-long-term physical performance after STN-DBS surgery, the effect of STN-DBS surgery on axial symptoms was said to be poor [7, 32]. However, due to the scarcity of studies that have investigated the short-term effects of STN-DBS on physical function, it is not possible to provide a definitive answer about the short-term effects of STN-DBS monotherapy on physical performance. A double-blind study reported that STN-DBS had no significant effect on postural stability assessed with Mini-BESTest, whereas STN-DBS improved overall motor performance assessed by UPDRS motor examination [33]. If stimulation of STN-DBS had no direct effect on postural stability, postoperative rehabilitation with stimulation would have some positive effect on the learning of postural stability. In this study, since the effects of rehabilitation after STN-DBS surgery were not directly compared with those of rehabilitation combined with drug treatment, it is difficult to give a clear answer regarding the difference in effects. However, there is a possibility that the combined use of rehabilitation after STN-DBS surgery can be expected to have an effect on the physical function that exceeds the maximum physical function during preoperative drug treatment, and we can recommend the use of postoperative rehabilitation in a clinical setting.

In the present study, there was no change in gravitational sway during open-eye standing, but gravitational sway during eye closure was significantly increased at discharge. Previous studies have reported a concern that STN-DBS was less effective in reducing center of gravity sway, namely, static balance [34], and the present results agreed with this. Another study reported the opposite result to the present study, that STN-DBS reduces center of gravity sway but does not improve dynamic balance [32]. In the present study, center of gravity sway was increased during eye closure even though overall balance function was improved. This suggests that postoperative rehabilitation with STN-DBS was not effective for keeping quiet standing without visual sensory input, and this might reflect acutely changed sensory integration of proprioceptive and vestibular inputs. It is unclear whether these changes represent a novel motor learning process or just a short-term postoperative complication, and further research is needed.

### 4.1. Limitations

There are many limitations to this study. One is the weakness of the study design. Because medical guidelines recommend rehabilitation of patients with PD, it would be unethical to design a study in which postoperative rehabilitation of STN-DBS patients was not performed. Therefore, it was difficult to design a case-control study design with a control group. In addition, because this study had no control group, it was difficult to investigate the effects of STN-DBS treatment and postoperative neurological physical therapy separately. Furthermore, it is difficult to determine the long-term effects of postoperative rehabilitation. In the present study, there was an improvement in trunk function and lower extremity muscle strength, as well as balance function, but a direct causal relationship between trunk function and lower extremity muscle strength and improvement in balance function cannot be directly addressed due to the study design. In addition, further study is needed to determine what kind of postoperative physical therapy is most appropriate for the patient. In light of the above, case-control studies by intervention type and randomized, controlled trials with target groups need to be performed in the future to better understand the effects of postoperative rehabilitation.

## 5. Conclusion

The combination of STN-DBS postoperative stimulation therapy with rehabilitation may provide short-term improvements in physical function that cannot be achieved with preoperative drug therapy alone. In addition, the effects of postoperative rehabilitation may also be reflected in postural stability, which has been considered to be difficult to achieve with STN-DBS.

## Figures and Tables

**Table 1:** Demographic data of the participants.

	Total (*n* = 60)	PI ≥ 3 (*n* = 18)	PI < 3 (*n* = 42)	*p* value between PI ≥ 3, PI < 3
Age, years	60.7 (8.9)	61.2 (9.9)	60.5 (8.6)	0.81
Sex, female/male	28 (47%)/32 (53%)	13 (72%)/5 (28%)	15 (36%)/27 (64%)	—
Duration of disease, years	12.2 (4.6)	12.8 (4.3)	11.9 (4.8)	0.49
Duration of medication, years	10.6 (4.1)	10.1 (4.1)	11.9 (4.0)	0.12
H & Y stage (on-state)	2.4 (0.7)	2.8 (0.5)	2.2 (0.7)	<0.001^*∗∗*^
MDS-UPDRS part III score of preoperation (on-state)	18.1 (8.6)	24.9 (6.9)	15.1 (7.6)	<0.001^*∗∗*^
MDS-UPDRS part III 12 postural stability score of preoperation (on-state)	1.2 (1.3)	3.0 (0)	0.4 (0.6)	<0.001^*∗∗*^
Final stimulation setting				
Pulse, microseconds	58.8 (4.9)	57.2 (7.3)	59.4 (3.3)	0.24
Hz	131.2 (6.5)	131.0 (7.1)	131.2 (6.3)	0.90
mA	1.8 (0.5)	1.8 (0.5)	1.8 (0.5)	0.97
Preoperative LDD	740 (272)	806 (275)	712 (269)	0.22
Discharge LDD	332 (218)	300 (227)	345 (216)	0.46
Preoperative LEDD	1464 (490)	1549 (456)	1439 (496)	0.43
Discharge LEDD	847 (456)	846 (502)	844 (444)	0.99
Duration of hospitalization, days	25.2 (16.9)	24.7 (14.3)	25.4 (18.1)	0.88

Data are means (SD), *n* (%). abbreviations: H & Y, Hoehn and Yahr; MDS-UPDRS, movement disorders society-unified Parkinson's disease rating scale; PI, MDS-UPDRS part III 12 postural instability score; LDD, levodopa daily dose; LEDD, levodopa equivalent daily dose.

**Table 2:** Results of post-operative rehabilitation with STN-DBS.

		PRE	POST	DISC	*p* value ANOVA	*F*value	*p* value (PRE-POST)	*p* value (PRE-DISC)	*p* value (POST-DISC)
Mini-BESTest		19.8 (5.1)	20.3 (4.8)	23.1 (4.3)	<0.0001^*∗∗*^	32.2	N.S	^*∗∗*^	^*∗∗*^
TUG seconds		10.4 (4.0)	11.3 (6.0)	9.7 (3.3)	0.053	3.0	N.S	N.S	N.S
TUG steps		17.1 (7.2)	17.1 (6.5)	14.7 (3.7)	0.003^*∗∗*^	6.1	N.S	^*∗∗*^	^*∗∗*^
TUG-cog seconds		14.4 (7.3)	15.9 (14.1)	12.2 (4.7)	0.046^*∗*^	3.2	N.S	N.S	^*∗*^
TUG-cog steps		23.2 (15.8)	22.1 (10.9)	17.5 (5.3)	0.005^*∗∗*^	5.6	N.S	^*∗∗*^	^*∗*^
TIS		17.3 (3.8)	17.5 (3.1)	18.8 (2.5)	<0.0001^*∗∗*^	11.8	N.S	^*∗∗*^	^*∗∗*^
10 times toe-tapping time (seconds)		4.4 (1.4)	4.0 (0.8)	3.8 (0.8)	0.0008^*∗∗*^	7.7	^*∗*^	^*∗∗*^	N.S
StrengthErgo240 (Newton-meter)		143.1 (71.6)	142.9 (73.5)	157.9 (74.8)	<0.0001^*∗∗*^	15.0	N.S	^*∗∗*^	^*∗∗*^
Eyes opened standing	COP 95% range circle (mm^2^)	1175.5 (2206.3)	1077.9 (1274.7)	1293.3 (1906.7)	0.80	0.2	N.S	N.S	N.S
	COP path length (mm)	632 (587.9)	705.5 (654.7)	794.6 (687.0)	0.41	0.9	N.S	N.S	N.S
	COP average speed (mm/sec)	21.7 (18.6)	22.8 (21.0)	24.0 (21.0)	0.89	0.1	N.S	N.S	N.S
Eyes closed standing	COP 95% range circle (mm^2^)	1029.2 (1162.9)	1150.1 (1588.5)	2027.4 (2835.6)	0.011^*∗*^	4.7	N.S	^*∗∗*^	^*∗*^
	COP path length (mm)	880.9 (726.7)	926.0 (811.8)	1200.4 (1174.8)	0.04^*∗*^	3.4	N.S	^*∗*^	^*∗*^
	COP average speed (mm/sec)	22.8 (20.3)	28.2 (25.6)	36.7 (35.8)	0.007^*∗∗*^	5.2	N.S	^*∗*^	^*∗*^
LDD		740 (272)	767 (240)	332 (218)	<0.0001^*∗∗*^	109.2	N.S	^*∗∗*^	^*∗∗*^
LEDD		1464 (490)	1462 (516)	847 (456)	<0.0001^*∗∗*^	208.8	N.S	^*∗∗*^	^*∗∗*^

Data are mean (SD), ^*∗*^*p* < 0.05, ^*∗∗*^*p* < 0.01. abbreviations: PRE, preoperation; POST, postoperation; DISC, discharge; TUG, timed up and go test; TUG-cog, timed up and go test cognitive tasking; TIS, trunk impairment scale; mini-BESTest, mini-balance evaluation systems test; LDD, levodopa daily dose; LEDD, levodopa equivalent daily dose; COP, center of pressure.

**Table 3:** Multiple regression analysis for the prediction of mini-BESTest score improvements.

	*β*	*p* value	VIF	*R * ^2^
TUG steps	−0.544	<0.0001^*∗∗*^	1.41	0.4425
TIS	0.2859	0.023^*∗*^	1.07	

Data are means (SD), ^*∗*^*p* < 0.05, ^*∗∗*^*p* < 0.01. abbreviations: *β*, standardised partial regression coefficient; VIF, variance inflation factor; *R*^2^, multiple coefficient of determination; TUG, timed up and go test; TIS, trunk impairment scale.

## Data Availability

The data used in the current study to support the findings are included within this article.

## Abbreviations

STN-DBS: Subthalamic nuclear stimulation therapy
MDS-UPDRS: Movement disorder society-unified Parkinson's disease rating scale
Mini-BESTest: Mini-balance evaluation systems test
TUG: Timed up and go test
TIS: Trunk impairment scale
LDD: Levodopa daily dose
LEDD: Levodopa equivalent daily dose.
